# A KDM4-DBC1-SIRT1 Axis Contributes to TGF-b Induced Mesenchymal Transition of Intestinal Epithelial Cells

**DOI:** 10.3389/fcell.2021.697614

**Published:** 2021-09-22

**Authors:** Baoyu Chen, Wenhui Dong, Tinghui Shao, Xiulian Miao, Yan Guo, Xingyu Liu, Yifei Feng

**Affiliations:** ^1^Department of Pathophysiology, Nanjing Medical University, Nanjing, China; ^2^College of Life Sciences and Institute of Biomedical Research, Liaocheng University, Liaocheng, China; ^3^Department of General Surgery, The First Affiliated Hospital of Nanjing Medical University, Nanjing, China; ^4^The First School of Clinical Medicine, Nanjing Medical University, Nanjing, China

**Keywords:** intestinal fibrosis, epithelial-mesenchymal transition, SIRT1, transcriptional regulation, epigenetics, histone demethylase

## Abstract

Intestinal fibrosis is one of the common pathophysiological processes in inflammatory bowel diseases (IBDs). Previously it has been demonstrated that epithelial-mesenchymal transition (EMT) can contribute to the development of intestinal fibrosis. Here we report that conditional ablation of SIRT1, a class III lysine deacetylase, in intestinal epithelial cells exacerbated 2, 4, 6-trinitro-benzene sulfonic acid (TNBS) induced intestinal fibrosis in mice. SIRT1 activity, but not SIRT1 expression, was down-regulated during EMT likely due to up-regulation of its inhibitor deleted in breast cancer 1 (DBC1). TGF-β augmented the recruitment of KDM4A, a histone H3K9 demethylase, to the DBC1 promoter in cultured intestinal epithelial cells (IEC-6) leading to DBC1 *trans*-activation. KDM4A depletion or inhibition abrogated DBC1 induction by TGF-β and normalized SIRT1 activity. In addition, KDM4A deficiency attenuated TGF-β induced EMT in IEC-6 cells. In conclusion, our data identify a KDM4-DBC1-SIRT1 pathway that regulates EMT to contribute to intestinal fibrosis.

## Introduction

Inflammatory bowl diseases (IBDs) refer to a group of relapsing and heterogeneous intestinal disorders exemplified by Crohn’s disease and ulcerative colitis (Ananthakrishnan, 2015). Documented as early as the nineteenth century, IBDs are more pervasive in the industrialized nations although recent epidemiology studies have indicated that there is a growing trend in the prevalence and incidence of IBDs in the developing countries. For instance, the population affected by IBDs in China alone is projected to reach 1.5 million by 2025 creating considerable socioeconomic burdens (Kaplan, 2015). Patients diagnosed with IBDs typically present chronic abdominal pain, fever, abscess, and fistula; these patients, in most severe cases and in the long term, can develop intestinal cancer (D’Haens, 2010). IBDs are equally influenced by the genetics and the environment. Thus far, over 160 loci have been identified to confer altered susceptibility to the development of IBDs in humans (Jostins et al., 2012). On the other hand, smoking, diets, life style, pollution, and even personal hygiene have been reported as potential environmental risk factors for IBDs (Leong, 2010). Although IBDs are clinically considered as a gastroenterological disorder, extraintestinal systems, the immune system in particular, play pivotal roles (Digby-Bell et al., 2020).

Intestinal fibrosis is one of the most common complications observed in IBD patients that are characterized by excessive scarring in the region of inflammation (Latella et al., 2015). Limited or mild intestinal fibrosis in IBD patients may evade detection with no overt clinical manifestation. Severe cases of intestinal fibrosis, however, often require surgical resections (Rieder and Fiocchi, 2009). Myofibroblasts are believed to be the major mediator of intestinal fibrosis although their origins are often debated and remain controversial (Valatas et al., 2017). Resident fibroblasts, stellate cells, bone marrow stem cells, fibrocytes, pericytes, epithelial cells, and endothelial cells have been reported to contribute to the pool of mature myofibroblasts in the course of intestinal fibrosis (Lawrance et al., 2017). Kalluri and colleagues have shown, aided by genetic lineage tracing technique, that Villin^+^ intestinal epithelial cells could *trans*-differentiate into myofibroblasts, in a process known as epithelial-mesenchymal transition (EMT), thereby contributing to intestinal fibrosis in mice (Flier et al., 2010). EMT and the closely related EndMT (endothelial-mesenchymal transition) are conserved pathobiological processes playing essential roles in organogenesis; EMT taking place post-developmentally is usually associated with such devastating diseases as malignant cancer and tissue fibrosis (Kalluri and Weinberg, 2009). During EMT, the cells shed the expression of epithelial signature genes (e.g., E-Cadherin encoded by *CDH1* and ZO-1 encoded by *TJP1*) in favor of mesenchymal marker genes (e.g., α-SMA encoded by *ACTA2* and collagen type I encoded by *COL1A1*).

Silent information regulator 1 (SIRT1) is the founding member of the mammalian sirtuin family of lysine deacetylases. SIRT1 regulates a wide range of pathobiological processes by differentially modulating the acetylation status of target proteins (Zschoernig and Mahlknecht, 2008). Mounting evidence suggests that SIRT1 generally plays a protective role in tissue fibrosis in multiple organs (Huang et al., 2014; Zerr et al., 2016; Bugyei-Twum et al., 2018; Li M. et al., 2018). Here we report that epithelial conditional ablation of SIRT1 exacerbates intestinal fibrosis in a TNBS-induced mouse model of colitis. SIRT1 activity, but not SIRT1 expression, is down-regulated during EMT owing to KDM4A mediated induction of DBC1 transcription.

## Materials and Methods

### Animals

All the animal protocols were reviewed and approved by the intramural Ethics Committee on Humane Treatment of Experimental Animals. The *Sirt1*^flox/flox^ strain (Li et al., 2017) was crossbred with the *Villin*-Cre strain (Sun et al., 2017) to generate intestinal epithelium-specific SIRT1 knockout (CKO) mice. Colitis was induced in the mice by intrarectal injection of TNBS (2.5% wt/vol) as previously described (Wirtz et al., 2017). The mice were euthanized 42 days after the onset of injection.

### Cell Culture

The rat intestinal epithelial cell IEC-6 (ATCC) was maintained in DMEM supplemented with 10% FBS. TGF-β was purchased from R&D. EX-527 and CP2 were purchased from Selleck. Small interfering RNAs were purchased from Dharmacon. Transient transfections were performed with Lipofectamine 2000 as previously described (Chen et al., 2021; Kong et al., 2021b; Liu et al., 2021a,b; Zhang et al., 2021).

### Protein Extraction, Immunoprecipitation, and Western Blot

Whole cell lysates were obtained by re-suspending cell pellets in RIPA buffer (50 mM Tris pH7.4, 150 mM NaCl, 1% Triton X-100) with freshly added protease inhibitor (Roche) as previously described (Hong et al., 2020; Dong et al., 2021; Kong et al., 2021a; Li et al., 2021). Nuclear proteins were extracted using the NE-PER Kit (Pierce) following manufacturer’s recommendation. Specific antibodies or pre-immune IgGs were added to and incubated with cell lysates overnight before being absorbed by Protein A/G-plus Agarose beads (Santa Cruz). Precipitated immune complex was released by boiling with 1X SDS electrophoresis sample buffer. Western blot analyses were performed with anti-SIRT1 (Santa Cruz, sc-74465), anti-α-SMA (Sigma, A2547), anti-E-Cadherin (Abcam, ab1416), anti-β-actin (Sigma, A2228), anti-DBC1 (Abcam ab215852), and anti-KDM4A (Abcam, ab191433) antibodies. For densitometrical quantification, densities of target proteins were normalized to those of β-actin. Data are expressed as relative protein levels compared to the control group which is arbitrarily set as 1.

### RNA Isolation and Real-Time PCR

RNA was extracted with the RNeasy RNA isolation kit (Qiagen). Reverse transcriptase reactions were performed using a SuperScript First-strand Synthesis System (Invitrogen) as previously described (Sun L. et al., 2020; Wu T. et al., 2020; Wu X. et al., 2020; Yang et al., 2020a,b). Real-time PCR reactions were performed on an ABI Prism 7500 system with the following primers: mouse *Cdh1*, 5′-AAGTGACCGATGATGATGCC-3′ and 5′-CTTCTCTGTCCATCTCAGCG-3′; mouse *Tjp1*, 5′-GATCCCTGTAAGTCACCCAGA-3′ and 5′-CTCCCTGCTT GCACTCCTATC-3′; mouse *Col1a1*, 5′-GACGCCATCAAGG TCTACTG-3′ and 5′-ACGGGAATCCATCGGTCA-3′; mouse *Acta2*, 5′-CTGAGCGTGGCTATTCCTTC-3′ and 5′-CTTCTG CATCCTGTCAGCAA-3′; rat *Cdh1*, 5′-GGAGAAGAAGAC CAGGACTTTG-3′ and 5′-GATGAAGTTCCCGATTTCATCAG-3′; rat *Acta2*, 5′-AGGATGCAGAAGGAGATCACAG-3′; and 5′-CTGGAAGGTAGATAGAGAAGCC-3′; rat *Tjp1*, 5′-GCAGTGTGAACATGGATTGAA-3′ and 5′-AGCCAATGCCT GACAGTTCT-3′; rat *Col1a1*, 5′-ATCTCCTGGTGCTGATG GAC-3′ and 5′-ACCTTGTTTGCCAGGTTCAC-3′; rat *Dbc1*, 5′-TCTCCAAGTCTCGCCTGTG-3′ and 5′-CTCTGTTGCC TCCAACCAGT-3′; mouse *Dbc1*. *Ct* values of target genes were normalized to the *Ct* values of housekeeping control gene (18s, 5′-CGCGGTTCTATTTTGTTGGT-3′ and 5′-TCGTCTTCGAAACTCCGACT-3′ for both human and mouse genes) using the ΔΔCt method and expressed as relative mRNA expression levels compared to the control group which is arbitrarily set as 1.

### Chromatin Immunoprecipitation (ChIP)

Chromatin Immunoprecipitation (ChIP) assays were performed essentially as described before (Coarfa et al., 2020; Hu et al., 2020; Jehanno et al., 2020; Maity et al., 2020; Mallik et al., 2020; Moon et al., 2020; Shen et al., 2020; Wang J. N. et al., 2020; Wang S. et al., 2020; Zhang et al., 2020; Zhao et al., 2020; Marti et al., 2021; Peng et al., 2021; Rashid et al., 2021). In brief, chromatin in control and treated cells were cross-linked with 1% formaldehyde. Cells were incubated in lysis buffer (150 mM NaCl, 25 mM Tris pH 7.5, 1% Triton X-100, 0.1% SDS, 0.5% deoxycholate) supplemented with protease inhibitor tablet and PMSF. DNA was fragmented into ∼200 bp pieces using a Branson 250 sonicator. Aliquots of lysates containing 200 μg of protein were used for each immunoprecipitation reaction with anti-acetyl H3 (Millipore, 06-599), anti-trimethyl H3K4 (Millipore, 07-473), anti-trimethyl H3K9 (Diagenode, C15410193), anti-trimethyl H3K27 (Millipore, 04-449), anti-trimethyl H4K20 (Millipore, 07-463), anti-KDM4A (Abcam, ab191433), anti-KDM4B (Bethyl Laboratories, A301-477A), anti-KDM4C (Bethyl Laboratories, A300-885A), or anti-KDM4D (Proteintech, 22591-1-AP) antibodies.

### SIRT1 Activity Assay

Whole cell lysates or tissue homogenates were prepared using the NETN buffer (20 mM Tris pH8.0, 100 mM NaCl, 1 mM EDTA, 0.5% NP-40) with freshly added protease inhibitor tablet and SIRT1 activities were measured with a SIRT1 assay kit (Sigma) according to vender’s recommendations.

### Histology

Histologic analyses were performed essentially as described before (Chen et al., 2020a,b,c; Dong et al., 2020; Fan et al., 2020; Li et al., 2020a,b,c; Lv et al., 2020). Briefly, paraffin-embedded sections were stained with picrosirius red (Sigma-Aldrich) or Masson’s trichrome (Sigma-Aldrich) according to standard procedures. Pictures were taken using an Olympus IX-70 microscope (Olympus, Tokyo, Japan).

### Statistical Analysis

One-way ANOVA with *post-hoc* Scheffe analyses were performed by SPSS software (IBM SPSS v18.0, Chicago, IL, United States). Unless otherwise specified, values of *p* < 0.05 were considered statistically significant.

## Results

### Intestinal Epithelial Deletion of SIRT1 Exacerbates TNBS Induced Intestinal Fibrosis

**FIGURE 1:**
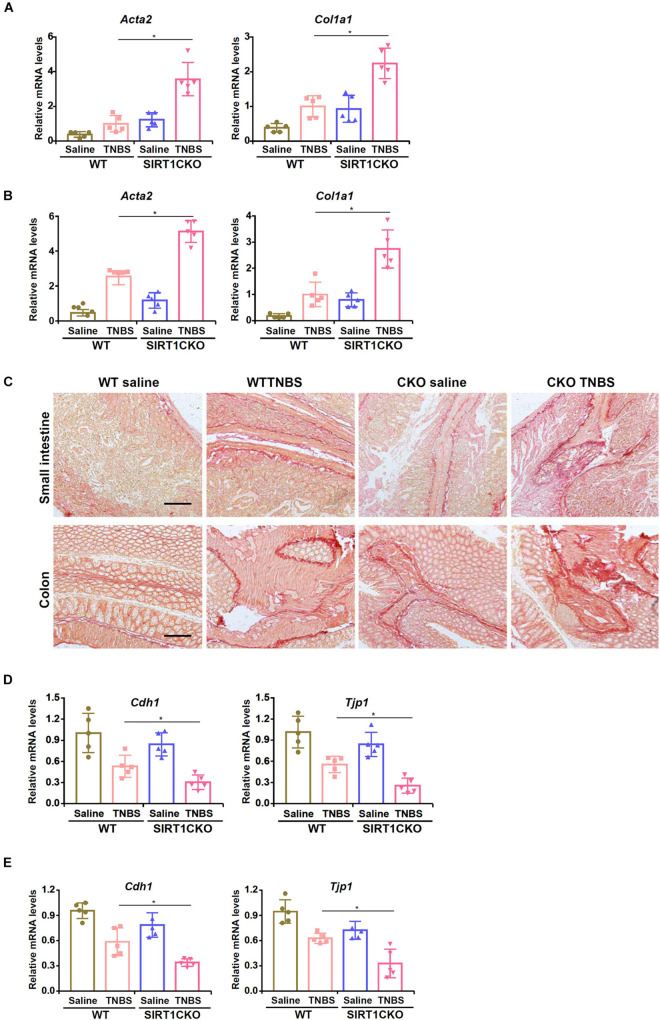
Intestinal epithelial deletion of SIRT1 exacerbates TNBS induced intestinal fibrosis. Intestine-conditional SIRT1 knockout (CKO) mice and wild type littermates were induced to develop colitis and intestinal fibrosis by TNBS injection. **(A)** Expression levels of pro-fibrogenic molecules in the small intestines were examined by qPCR. **(B)** Expression levels of pro-fibrogenic molecules in the colons were examined by qPCR. **(C)** Paraffin sections were stained with picrosirius red. Scale bar, 100 mm. **(D)** Expression levels of epithelial marker genes in the small intestines were examined by qPCR. **(E)** Expression levels of epithelial marker genes in the colons were examined by qPCR. *N* = 5 mice for each group.

To specifically delete SIRT1 in the intestinal epithelial cells, the *Sirt1*^f/f^ mice were crossed with the *Villin*-Cre mice. We first evaluated the development of intestinal fibrosis in the WT mice and the CKO mice. Quantitative PCR showed that TNBS injection significantly induced the expression of smooth muscle cell actin (*Acta2*) and collagen type I (*Col1a1*), two prototypical markers of myofibroblasts, in the small intestines (Figure 1A) and colons (Figure 1B). Compared to the WT mice, CKO mice displayed significantly augmented expression of pro-fibrogenic genes indicative of accelerated intestinal fibrosis. Picrosirius red staining confirmed that deposition of collagenous tissues in the small intestines and the colons was up-regulated in the mice following TNBS injection whereas the CKO mice exhibited more prominent intestinal fibrosis than the WT mice (Figure 1C).

Previously Kalluri and colleagues have demonstrated that epithelial-mesenchymal transition (EMT) plays a role in intestinal fibrosis in mice (Flier et al., 2010). Now that SIRT1 deficiency in epithelial cells resulted in up-regulation of mesenchymal marker genes (*Acta2* and *Col1a1*), we hypothesized that SIRT1 deficiency might promote EMT leading to accelerated loss of epithelial markers. Indeed, TNBS injection down-regulated the expression of E-Cadherin (*Cdh1*) and ZO-1 (*Tjp1*) in the intestines and the loss of *Cdh1* and *Tjp1* expression was more severe in the CKO mice than in the WT mice (Figures 1D,E).

Transforming growth factor (TGF-β) is considered one of the most potent inducers of EMT. TGF-β expression has been observed to elevate in animal models of IBD (Wengrower et al., 2004; Fichtner-Feigl et al., 2007) and in IBD patients (Li et al., 2013). Consistent with prior observations, TGF-β (Tgfb1) expression was up-regulated in the TNBS treated mice compared to the saline treated mice although the induction of TGF-β expression by TNBS was comparable between the WT and the CKO mice (Supplementary Figure 1). In a cell model of EMT wherein the rat intestinal epithelial IEC-6 cells were treated with TGF-β, over-expression of a wild type (WT) SIRT1, but not an enzymatically inactive (HY) SIRT1, partially reversed the effect of TGF-b (Supplementary Figure 2). Similarly, pre-treatment with a specific SIRT1 agonist (SRT1720) dose-dependently attenuated TGF-β induced EMT in IEC-6 cells (Supplementary Figure 3). Collectively, these data suggest that intestinal epithelial deletion of SIRT1 exacerbates TNBS induced intestinal fibrosis likely by promoting the EMT process.

### SIRT1 Activity Is Down-Regulated by TNBS *in vivo* and by TGF-β in Epithelial Cells *in vitro*

**FIGURE 2:**
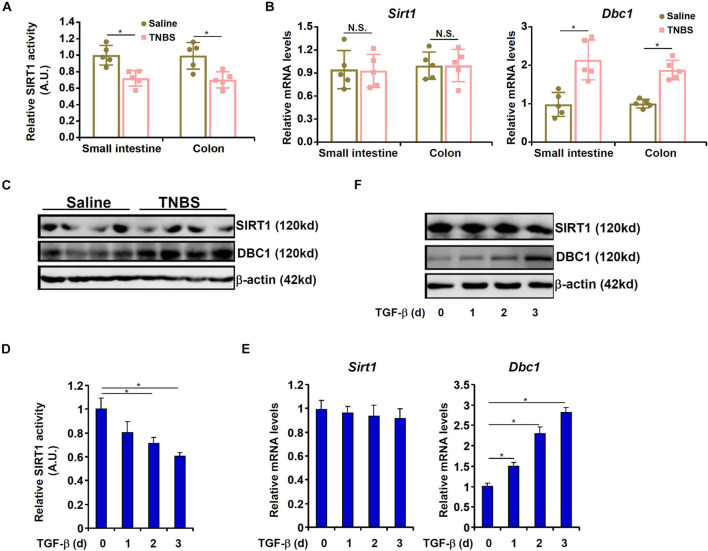
SIRT1 activity is down-regulated by TNBS *in vivo* and by TGF-β in epithelial cells *in vitro*. **(A–C)** Colitis was induced in C57B/6 mice by TNBS as described in section “Materials and Methods.” SIRT1 activity was determined by a colorimetric kit. Expression levels of SIRT1 and DBC1 were examined by qPCR and Western. *N* = 5 mice for each group. **(D–F)** IEC-6 cells were treated with TGF-β (2 ng/ml) and harvested at indicated time points. SIRT1 activity was determined by a colorimetric kit. Expression levels of SIRT1 and DBC1 were examined by qPCR and Western. All experiments were performed in triplicate wells and repeated three times. One representative experiment is shown.

We then asked whether SIRT1 expression and/or activity would be altered during intestinal fibrosis. As shown in Figure 2A, mice exposed to TNBS displayed significant down-regulation of SIRT1 activity in the intestines compared to the control mice. Of note, neither SIRT1 mRNA expression (Figure 2B) nor protein expression (Figure 2C) was affected by TNBS exposure in the mouse intestines suggesting that dampening of SIRT1 activity by TNBS may be attributed to a post-transcriptional mechanism. It has been previously reported that deleted in breast cancer 1 (DBC1) allosterically inhibits SIRT1 activity without altering its expression (Kim et al., 2008). Indeed, DBC1 expression was significantly up-regulated in the intestines in the TNBS-treated mice compared to the control mice (Figures 2B,C). To cement the role of TGF-β in DBC1 induction, we pre-treated the cells with a selective TβRI/II inhibitor (LY2109761). Indeed, blocking TGF-β engagement to its receptor by LY2109761 largely abrogated DBC1 up-regulation and prevented EMT in IEC-6 cells (Supplementary Figure 4A). Concordantly, suppression of SIRT1 activity was alleviated (Supplementary Figure 4B).

TGF-β treatment suppressed SIRT1 activity in a time course dependent manner without influencing SIRT1 expression in IEC-6 cells (Figures 2D–F). On the other hand, TGF-β stimulated DBC1 expression mirroring the suppression of SIRT1 activity in IEC-6 cells (Figures 2D–F). Furthermore, stronger association between DBC1 and SIRT1 was detected by co-immunoprecipitation in the TNBS-treated intestines (Supplementary Figure 5A) and in TGF-treated IEC-6 cells (Supplementary Figure 5B). Together, these data suggest that loss of SIRT1 activity, but not SIRT1 expression, likely due to increased DBC1 expression and enhanced DBC1-SIRT1 interaction may contribute to EMT in intestinal epithelial cells.

### DBC1 Contributes to TGF-β Induced EMT by Repressing SIRT1 Activity

**FIGURE 3:**
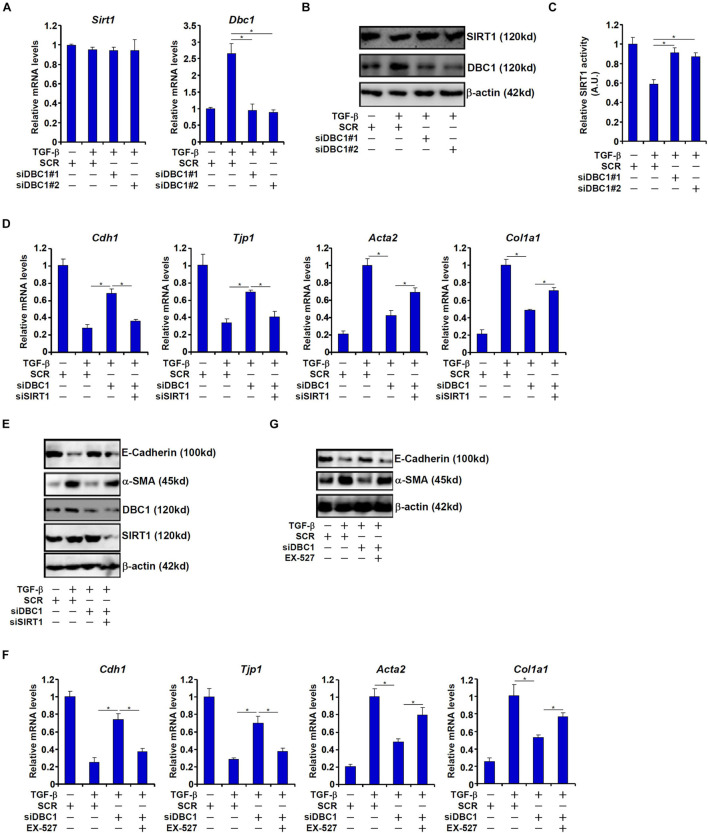
DBC1 contributes to TGF-β induced EMT by repressing SIRT1 activity. **(A–C)** IEC-6 cells were transfected with siRNAs targeting DBC1 or scrambled siRNAs followed by treatment with TGF-β (2 ng/ml). Expression levels of SIRT1 and DBC1 were examined by qPCR and Western. SIRT1 activity was determined by a colorimetric kit. **(D,E)** IEC-6 cells were transfected with indicated siRNAs followed by treatment with TGF-β (2 ng/ml) for 48 h. Gene expression levels were examined by qPCR and Western. **(F,G)** IEC-6 cells were transfected with indicated siRNAs followed by treatment with TGF-β (2 ng/ml) in the presence or absence of EX527 (10 mM) for 48 h. Gene expression levels were examined by qPCR and Western. All experiments were performed in triplicate wells and repeated three times. One representative experiment is shown.

In order to ascertain that DBC1 might mediate the suppressive effect of TGF-β on SIRT1 activity, siRNAs targeting DBC1 were transfected into IEC-6 cells. DBC1 depletion restored SIRT1 activity despite the presence of TGF-β (Figures 3A–C). Of note, DBC1 knockdown did not significantly alter SIRT1 expression consistent with its role as an allosteric inhibitor of SIRT1.

Next, we asked whether DBC1 could contribute to TGF-β induced EMT. As shown in Figures 3D,E, DBC1 knockdown attenuated down-regulation of *Cdh1* expression and *Tjp1* expression and up-regulation of *Acta2* expression and *Col1a1* expression in TGF-β treated IEC-6 cells. Simultaneous depletion of SIRT1, however, blocked the anti-EMT effect of DBC1 knockdown, suggesting that DBC1 may rely on its function as a SIRT1 inhibitor to regulate EMT. Similarly, SIRT1 inhibition by EX-527 largely abrogated the normalization of epithelial/mesenchymal signature genes by DBC1 knockdown and enabled TGF-β to stimulate EMT in IEC-6 cells (Figures 3F,G).

### TGF-β Promotes KDM4A Recruitment to the DBC1 Promoter

**FIGURE 4:**
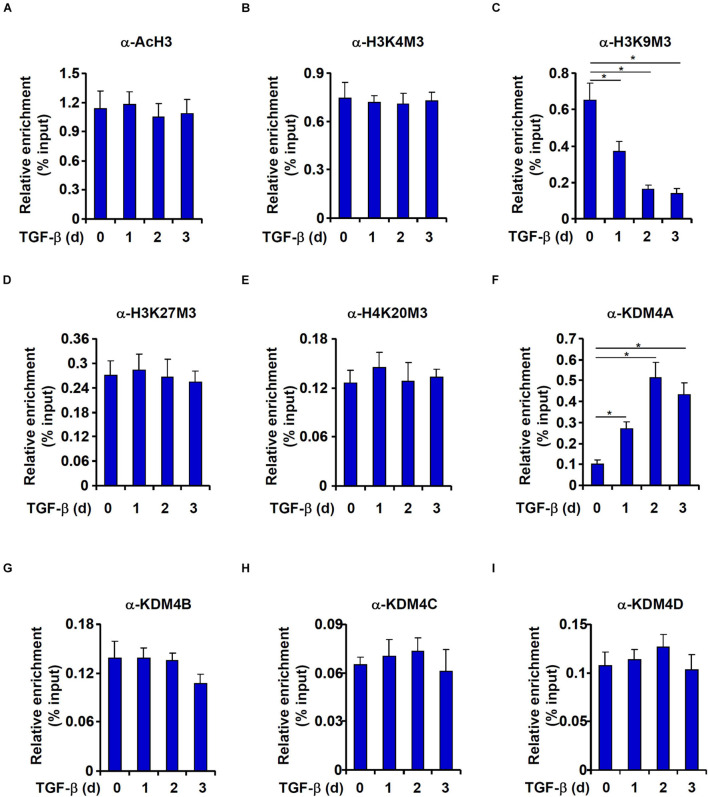
TGF-β promotes KDM4A recruitment to the DBC1 promoter. **(A–I)** IEC-6 cells were treated with TGF-β(2 ng/ml) and collected at indicated time points. ChIP assays were performed anti-acetyl H3, anti-trimethyl H3K4, anti-trimethyl H3K9, anti-trimethyl H3K27, anti-trimethyl H4K20, anti-KDM4A, anti-KDM4B, anti-KDM4C, or anti-KDM4D. All experiments were performed in triplicate wells and repeated three times. One representative experiment is shown.

We then performed ChIP assays to explore the epigenetic mechanism whereby TGF-β activates DBC1 expression. High levels of acetylated histone H3 (Figure 4A) and trimethylated H3K4 (Figure 4B), two typical markers for actively transcribed chromatin, were detected on the DBC1 promoter under basal conditions and were not noticeably up-regulated by TGF-β treatment. On the contrary, levels of trimethylated H3K9, a marker for silenced chromatins, were down-regulated by TGF-b treatment (Figure 4C). By comparison, trimethylated H3K27 (Figure 4D) and trimethylated H4K20 (Figure 4E), both reported to be associated with transcriptional repression, were not significantly altered by TGF-β treatment.

Because removal of trimethyl H3K9 from the DBC1 promoter was observed in TGF-β induced EMT process, we hypothesized that TGF-β treatment might enhance the occupancies of specific histone H3K9 demethylases. Indeed, KDM4A association with the DBC1 promoter was enhanced by TGF-β treatment with a kinetics closely mirroring that of H3K9M3 removal (Figure 4F). In contrast, occupancies of KDM4B (Figure 4G), KDM4C (Figure 4H), or KDM4D (Figure 4I) on the DBC1 promoter were not impacted by TGF-β treatment.

### KDM4A Mediates TGF-β Induced DBC1 Transcription to Promote EMT

**FIGURE 5:**
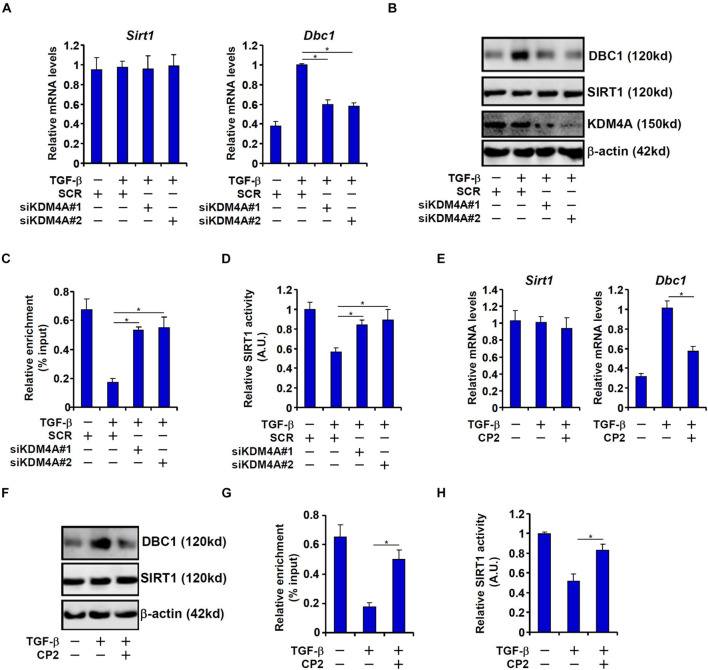
KDM4A mediates TGF-β induced DBC1 transcription. **(A–D)** IEC-6 cells were transfected with siRNAs targeting KDM4A or scrambled siRNAs followed by treatment with TGF-β (2 ng/ml). DBC1 expression levels were examined by qPCR and Western. ChIP assay was performed with anti-trimethyl H3K9. SIRT1 activity was determined by a colorimetric kit. **(E–H)** IEC-6 cells were treated with TGF-β (2 ng/ml) in the presence or absence of CP2 (1 μM). DBC1 expression levels were examined by qPCR and Western. ChIP assay was performed with anti-trimethyl H3K9. SIRT1 activity was determined by a colorimetric kit. All experiments were performed in triplicate wells and repeated three times. One representative experiment is shown.

To confirm that KDM4A was responsible for DBC1 induction by TGF-β and its relevance to EMT, the following experiments were performed. KDM4A knockdown by two separate pairs of siRNAs dampened the DBC1 induction in TGF-β treated IEC-6 cells at mRNA (Figure 5A) and protein (Figure 5B) levels. Consistent with the changes in DBC1 expression, a concomitant change in H3K9M3 levels was detected on the DBC1 promoter (Figure 5C). As a result, inhibition of SIRT1 activity was partially rescued (Figure 5D). In a second set of experiments, CP2 was used to inhibit KDM4A activity (Kawamura et al., 2017). KDM4A inhibition similarly antagonized DBC1 induction (Figures 5E,F) and removal of H3K9M3 (Figure 5G) by TGF-β. Consequently, CP2 treatment led to a partial normalization of SIRT1 activity (Figure 5H).

**FIGURE 6:**
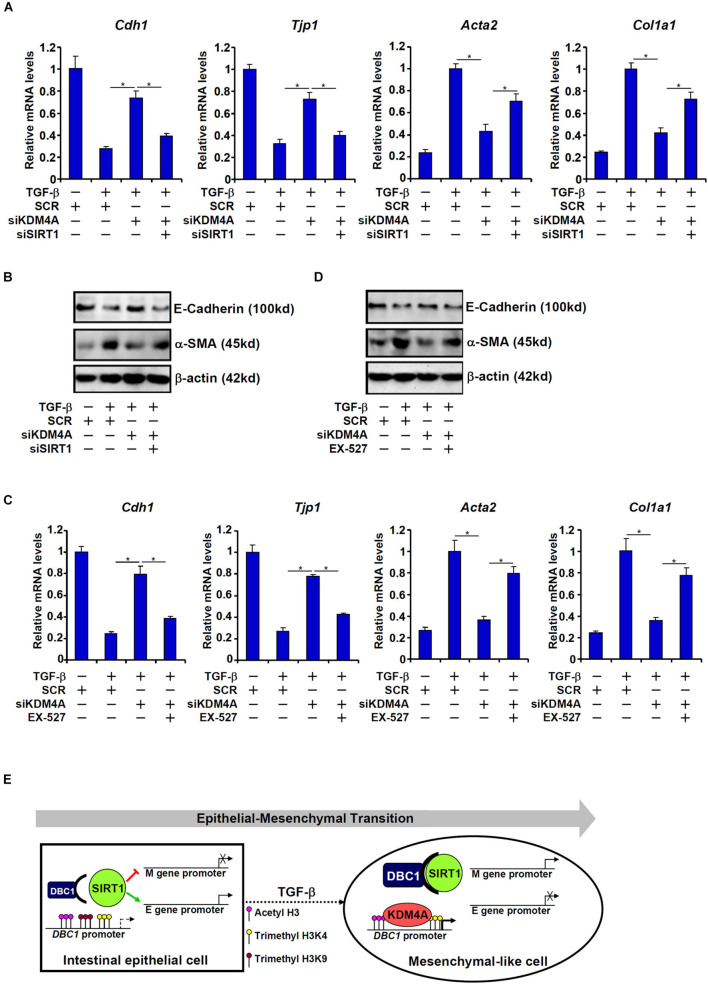
KDM4A silencing or inhibition attenuates TGF-β induced EMT in a SIRT1-dependent manner. **(A,B)** IEC-6 cells were transfected with indicated siRNAs followed by treatment with TGF-β (2 ng/ml) for 48 h. Gene expression levels were examined by qPCR and Western. **(C,D)** IEC-6 cells were transfected with indicated siRNAs followed by treatment with TGF-β (2 ng/ml) in the presence or absence of EX527 (10 μM) for 48 h. Gene expression levels were examined by qPCR and Western. All experiments were performed in triplicate wells and repeated three times. One representative experiment is shown. **(E)** A schematic model.

On the other hand, KDM4A knockdown, similar to DBC1 knockdown, resulted in up-regulation of *Cdh1* expression and *Tjp1* expression and down-regulation of *Acta2* expression and *Col1a1* expression, a trend reversed by simultaneous SIRT1 depletion (Figures 6A,B). SIRT1 inhibition by EX-527 similarly circumvented KDM4A deficiency to allow TGF-β to down-regulate *Cdh1* expression and *Tjp1* expression and up-regulate *Acta2* expression and *Col1a1* expression (Figures 6C,D).

## Discussion

Intestinal fibrosis, one of the most common complications of IBDs, remains a daunting challenge due to the lack of effective therapeutic solutions. Previous investigations have suggested that the developmentally conserved EMT process plays a role in the pathogenesis of intestinal fibrosis (Lovisa et al., 2019). Here we provide evidence that supports the involvement of a KDM4A-DBC1-SIRT1 axis in the regulation of EMT potentially contributing to intestinal fibrosis.

We show here that down-regulation of SIRT1 activity, but not SIRT1 expression, is a determining factor in TNBS-induced intestinal fibrosis. Further, our data suggest that DBC1 is responsible for the suppression of SIRT1 activity in intestinal epithelial cells in the process of EMT, which alludes to the possibility that DBC1 might drive EMT and intestinal fibrosis. It is noteworthy that this hypothesis is buttressed by several indirect pieces of evidence. First, Gao et al. (2015) have reported that mice with systemic DBC1 deletion are resistant to colitis although the underlying mechanism is proposed to be mediated by inhibition of Foxp3, a key anti-inflammatory transcription factor, by DBC1 in regulatory T cells; it remains undetermined whether the mice with epithelial-specific deletion of DBC1 would phenocopy the systemic DBC1 KO mice in the pathogenesis of intestinal fibrosis. Second, DBC1 activates the expression of MMP7, a mesenchymal cell marker, to pivot breast cancer cells to a more malignant phenotype by interacting with the zinc finger transcription factor ZFN326 (Yu et al., 2018). Third, a recent investigation by Kim et al. (2018) has revealed a role for DBC1 in the regulation of colorectal cancer metastasis; DBC1 directly interacts with β-catenin and stabilizes the β-catenin/LEF1 complex to activate the transcription of metastasis-associated in colon cancer 1 (MACC1), an EMT-related molecule. It would be of great interest to, along this line of investigations, further delineate the role of DBC1 in intestinal fibrosis.

Our data suggest that the lysine demethylase KDM4A can contribute to TGF-β induced EMT by stimulating DBC1 transcription to inhibit SIRT1 activity. KDM4A knockdown or pharmaceutical inhibition normalizes DBC1 expression and SIRT1 activity leading to stagnation of EMT. This is consistent with a report by Ishiguro et al. (2017) showing that pharmaceutical inhibition of KDM4A alleviates colitis in mice likely through the IL-6 signaling. However, these observations do not entirely foreclose the possibility that KDM4A may contribute to intestinal fibrosis via alternative mechanisms. Pezone et al. (2020) have recently reported that KDM4A can be recruited to the *SNAI1* promoter and activate *SNAI1* transcription in response to TGF-β stimulation in mammalian epithelial cells. The E-box binding transcriptional repressor SNAIL, which is encoded by *SNAI1*, subsequently binds to the promoters of epithelial signature genes to repress transcription thus initiating the EMT process. Alternatively, Sun S. et al. (2020) have shown that KDM4A can directly bind to the promoter and activate the transcription of Myc, a master regulator of EMT, in lung cancer cells. It should be pointed out that our proposed model (Figure 6E) and any alternative scenarios alluded to by other investigations are not mutually exclusive. For instance, it has been observed that SIRT1 can directly interact with Myc and deacetylate Myc leading to Myc degradation by the ubiquitin-proteasome system (Yuan et al., 2009). Alternatively, SIRT1 has been shown to deacetylate SNAIL and modulate SNAIL activity in lung cancer cells (Yang et al., 2021). Further analysis is warranted to delineate the functional interplay between KDM4A and SIRT1.

There are still major limitations associated with the present study. First, although we focused on SIRT1 as a regulator of TGF-induced EMT and intestinal fibrosis, it is highly likely that a group of factors, rather than a single factor alone (e.g., SIRT1), collectively mediate the TGF-β effect. For instance, the E-box recognizing family of transcriptional repressors, including Snail, Slug, Twist, and Zeb, are well characterized mediators of EMT in various systems (Lamouille et al., 2014). Recently, Scharl et al. (2013) have observed that Snail1 appears to play a critical role in TGF induced EMT in monolayer intestinal epithelial cells and IEC spheroids. However, the potential contribution by these proteins and the model as supported by our data are not necessarily mutually exclusive because SIRT1 has been reported to influence the activities of these transcription factors via protein-protein interactions or altering expression levels (Choupani et al., 2018). Additional investigation is warrant to sort out the entanglement of these EMT factors. Second, our proposal that a KDM4A-DBC1 axis mediates TGF-β induced SIRT1 repression remains to be validated *in vivo*. This is partly due to the lack of appropriate model animals (for instance, mice harboring epithelial-specific KDM4A/DBC1/TβRI/II deletion). Further compounding the issue is the fact that despite the development of a plethora of TGF-β targeting compounds (Teixeira et al., 2020), none have been demonstrated to confer protective effects on intestinal fibrosis. Therefore, a large disconnect exists between the *in vitro* observations and the *in vivo* pathology. Third, the precise mechanism whereby SIRT1 regulates EMT and intestinal fibrosis awaits further investigation. It is generally agreed that SIRT1 relies on its activity as a lysine deacetylase to participate in pathophysiological processes, a notion supported by our observation (Supplementary Figure 3). However, SIRT1 can use both histones and non-histone factors as substrates to orchestrate downstream events. For instance, pro-EMT/fibrogenic transcription factors, including NF-κB (Li X. et al., 2018), β-catenin (Simic et al., 2013), c-Myc (Yuan et al., 2009), and SMAD (Chen et al., 2014), can all be modulated by SIRT1-mediated lysine deacetylation. In addition, SIRT1 is able to bind to and directly deacetylate histones H3/H4 surrounding the pro-fibrogenic gene promoters (Guo et al., 2017). It would be helpful to exploit transcriptomic and proteomic tools to comprehensively profile the influence of SIRT1 on the acetylation status of both histones and non-histone factors in the context of EMT and intestinal fibrosis. Finally, although we propose here that DBC1 might contribute to TGF induced EMT and intestinal fibrosis via SIRT1 suppression, the role of other DBC1-interacting factors cannot be fully excluded. For instance, Chini et al. (2010) have identified HDAC3, a class I lysine deacetylase, as a binding partner for DBC1; binding of DBC1 to HDAC3 inhibits HDAC3 activity. A string of recent reports have implicated HDAC3 as an important regulator of EMT and/or tissue fibrosis (Ning et al., 2021). Alternatively, interaction with DBC1 stabilizes the nuclear receptor Rev-erbα (Chini et al., 2013). Recent studies have shown that Rev-erbaα is an emerging target for tissue fibrosis (Li et al., 2014; Welch et al., 2017). It is plausible that a panel of DBC1-interacting proteins, rather than SIRT1 alone, collectively determine its function as a modulator of EMT and/or intestinal fibrosis, which certainly deserves to be thoroughly investigated in future studies.

In summary, evidence has been mounting that targeting SIRT1 may be considered as a viable approach to treat tissue fibrosis. Indeed, small-molecule compounds that boost SIRT1 activity has been exploited to mitigate tissue fibrosis with success at least in model animals (Ponnusamy et al., 2015; Liu et al., 2017; Yu et al., 2017; Chu et al., 2018). Our data as reported here reinforce the notion that suppression of SIRT1 activity represents a key pathophysiological event in the pathogenesis of intestinal fibrosis and argue for the screening of small-molecule SIRT1 agonists in the intervention of intestinal fibrosis.

## Data Availability Statement

The raw data supporting the conclusions of this article will be made available by the authors, without undue reservation.

## Ethics Statement

The animal study was reviewed and approved by Nanjing Medical University Ethics Committee on Humane Treatment of Laboratory Animals.

## Author Contributions

YF conceived the project. BC, WD, TS, XM, YG, and XL designed and performed the experiments, and collected data. All authors contributed to the drafting and editing of the manuscript.

## Conflict of Interest

The authors declare that the research was conducted in the absence of any commercial or financial relationships that could be construed as a potential conflict of interest.

## Publisher’s Note

All claims expressed in this article are solely those of the authors and do not necessarily represent those of their affiliated organizations, or those of the publisher, the editors and the reviewers. Any product that may be evaluated in this article, or claim that may be made by its manufacturer, is not guaranteed or endorsed by the publisher.
