# Supplementary material for: A KDM4-DBC1-SIRT1 Axis Contributes to TGF-b Induced Mesenchymal Transition of Intestinal Epithelial Cells
Source: Front Cell Dev Biol. 2021 Sep 22;9:697614. doi: 10.3389/fcell.2021.697614 (PMC8493255; doi:10.3389/fcell.2021.697614)
Supplement: Supplementary file 1 [file Table_1.DOC]

**Chen B et al: A KDM4-DBC1-SIRT1 axis contributes to TGF-β induced mesenchymal transition of intestinal epithelial cells**

**Online supplementary figures**

**Fig.S1:** Intestine-conditional SIRT1 knockout (CKO) mice and wild type littermates were induced to develop colitis and intestinal fibrosis by TNBS injection. Tgfb1 expression levels in the small intestines (A) and colons (B) were examined by qPCR.

**Fig.S2:** IEC-6 cells were infected with lentivirus carrying either wild type or inactive SIRT1 followed by treatment with or without TGF-β (2ng/ml) for 3 days. (**A**) Efficiencies of SIRT1 over-expression were verified by Western. (**B**) Gene expression levels were examined by qPCR.

**Fig.S3:** IEC-6 cells were treated with TGF-β (2ng/ml) in the presence or absence of SRT1720 (5μM, 15μM) for 3 days. Gene expression levels were examined by qPCR.

**Fig.S4:** IEC-6 cells were treated with TGF-β (2ng/ml) in the presence or absence of LY2109761 (1μM) for 3 days. (**A**) Gene expression levels were examined by qPCR. (**B**) SIRT1 activity was determined by a colorimetric kit.

**Fig.S5:** (**A**) Small intestines were isolated from the saline-treated mice and the TNBS-treated mice. Immunoprecipitation was performed with anti-SIRT1 using whole tissue lysates. (**B**) IEC-6 cells were treated with TGF-β (2ng/ml) and harvested at indicated time points. Immunoprecipitation was performed with anti-SIRT1 using whole cell lysates.
